# Oral Appliances in Obstructive Sleep Apnea

**DOI:** 10.3390/healthcare7040141

**Published:** 2019-11-08

**Authors:** Marijke Dieltjens, Olivier M. Vanderveken

**Affiliations:** 1 Department of Translational neurosciences, Faculty of Medicine and Health Sciences, University of Antwerp, 2610 Wilrijk, Belgium; marijke.dieltjens@uza.be; 2 Special Dentistry Care, Antwerp University Hospital, 2650 Edegem, Belgium; 3 ENT, Head and Neck Surgery, Antwerp University Hospital, 2650 Edegem, Belgium; 4 Multidisciplinary Sleep Disorders Centre, Antwerp University Hospital, 2650 Edegem, Belgium

**Keywords:** mandibular advancement therapy, treatment, sleep-disordered breathing

## Abstract

Oral appliance therapy is increasingly prescribed as a non-invasive treatment option for patients diagnosed with obstructive sleep apnea. The custom-made titratable mandibular advancement devices (MAD) are the recommended type of oral appliances. Mandibular advancement devices are efficacious in reducing the severity of obstructive sleep apnea, however, only to a lesser extent than standard therapy using continuous positive airway pressure (CPAP). Although oral appliance therapy is known to reduce the severity of obstructive sleep apnea in most of the patients, one out of three patients still show negligible improvement under MAD therapy. Therefore, the selection of the appropriate candidates for this therapy is imperative and several upfront prediction tools are described. Overall, the health outcome of mandibular advancement device therapy is similar to that of CPAP, probably due to the inferior compliance of CPAP compared to MAD therapy, resulting in similar clinical effectiveness.

## 1. Introduction

Obstructive sleep apnea (OSA) is an increasingly common disorder, affecting approximately 17% of adult women and 34% of men [1]. The main pathophysiological feature of OSA is repetitive narrowing (hypopnea) or closure (apnea) of the upper airway (UA) during sleep, causing intermittent hypoxia, intrathoracic pressure swings, sympathetic surges, and sleep fragmentation [2]. Due to these perturbations, OSA is linked to a range of harmful sequelae: excessive daytime sleepiness, fatigue, an impaired cognitive performance, a reduced quality of life, an increased risk of occupational and traffic accidents [3], metabolic disturbances [4], hypertension [5], cardio- and cerebrovascular morbidity, and OSA-related mortality [6].

Due to the high prevalence, as well as the individual and socioeconomic healthcare issues related to OSA, the effective management of this chronic disorder is imperative. The standard treatment for patients with moderate to severe OSA is continuous positive airway pressure (CPAP), applying pressurized air throughout the respiratory cycle to keep the upper airway patent [7]. Although CPAP is highly efficacious in reducing the severity of OSA, the clinical effectiveness is often compromised by low patient acceptance and suboptimal adherence [8].

Oral appliance therapy is increasingly prescribed as a non-invasive treatment option for patients with OSA. Oral appliances are indicated for use in patients with mild to moderate OSA who prefer oral appliance therapy to CPAP, who do not respond to CPAP, are not appropriate candidates for CPAP, or who fail treatment attempts with CPAP [9].

## 2. Types of Oral Appliances

Oral appliances can be divided into three main categories, based on their mode of action. First, soft palate lifters aim to reduce vibrations from the soft palate by elevating both the soft palate and uvula. However, there is little evidence regarding their effectiveness [10,11]. Second, tongue retaining devices (TRD) use a suction pressure to hold the tongue in a forward position during sleep and thereby prevent the tongue from falling back into the pharyngeal airway [12,13]. The third category is the oral appliances advancing the mandible and the attached tongue during the night, known as mandibular advancement devices (MADs), mandibular advancement appliances (MAAs), mandibular repositioning appliances (MRAs), or mandibular advancement splints (MASs) [12]. The MAD is the most common type of oral appliance therapy used for the treatment of OSA [14]. The mechanism of action of the MAD is usually assumed to cause the enlargement of the cross-sectional upper airway dimensions by anterior displacement of the mandible and the attached tongue, resulting in improved upper airway patency [15,16,17].

There is a huge variety of commercially available MADs, all with different design features [18]. MADs can be custom-made or prefabricated thermoplastic devices. Custom-made appliances are fabricated from dental casts of the patient’s dentition and bite registration by the dentist. A lower cost alternative are the thermoplastic or “boil and bite” appliances, that can be fitted without the need for plaster casts or bite registrations. A randomized controlled trial comparing the efficacy of a custom-made appliance with a thermoplastic device provided primary evidence that a custom-made MAD is more efficacious in reducing the OSA severity than a prefabricated MAD [19]. Therefore, custom-made appliances are recommended over prefabricated devices made out of thermoplastic materials [19].

**Figure 1:**
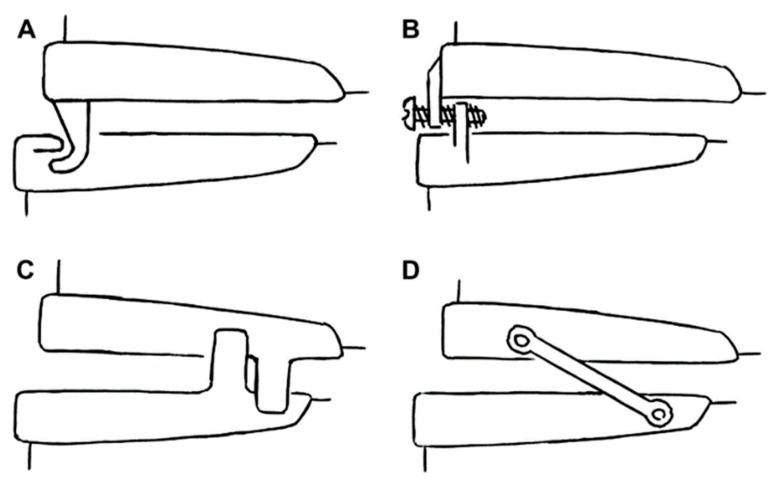
Schematic overview of titratable, duobloc mandibular advancement device (MAD) designs used in current clinical practice: (**A**) MAD with an anteriorly articulating component that allows the MAD for adjustment of the appliance; (**B**) MAD with attachments for the adjustment of mandibular protrusion in the frontal teeth area; (**C**) MAD with two lateral positioning attachments that permit incremental protrusion of the mandible; (**D**) MAD with lateral telescopic rods that force the mandible into an anterior position.(Figure published in Dieltjens et al., 2012 [31]).

Furthermore, the concept of custom-made MADs has evolved from the “monobloc” type of device where upper and lower parts are rigidly connected, towards the current “duobloc” types. The rigid monobloc MADs restricts mandibular movements, which sometimes produces temporomandibular discomfort. The so-called titratable MADs allow for fine-tuning of the mandibular advancement as the upper and lower parts are separate but dynamically interconnected [20,21]. Several titratable MADs with different basic advancement mechanism are tested in the literature and are summarized in Figure 1.

A randomized controlled trial demonstrated that a thermoplastic heat-molded titratable MAD was non-inferior in the short-term to a custom-made acrylic MAD. Therefore, such a thermoplastic titratable MAD can be used as a simple, cheap, and ready to use method to identify patients likely to benefit from long-term MAD therapy [22].

According to the literature, the amount of protrusion constitutes a key factor in optimizing MAD efficacy, although more protrusion does not always yield better results [23]. Therefore, the optimal mandibular protrusion for MAD therapy needs to be determined in the individual patient and thereafter adjusted in terms of tolerability versus efficacy [24]. However, up until now, no proven standard is available on how to determine this optimal MAD protrusion. Most outcome studies on MAD therapy are using a so-called “subjective titration protocol,” relying on both the physical limits of the patient’s mandibular protrusion and the self-reported evolution of symptoms, such as snoring and/or daytime sleepiness [25,26,27,28]. However, such subjective improvement in symptoms may not provide the most accurate indicator for efficient titration of the MAD: it may result in a suboptimal treatment outcome, since the reduction of the subjective complaints may encourage a premature interruption of the titration [29,30]. So, at this stage, MAD titration remains a “trial and error” approach [31]. In an approach analogous to a CPAP titration night, the mandible can be progressively advanced during sleep, each time respiratory events occur. A so-called “remotely controlled mandibular positioner” (RCMP) has been applied in overnight sleep studies to prospectively determine the optimal mandibular protrusion for MAD treatment in individual patients [24,31,32,33,34]. Literature has shown a greater reduction in OSA severity after RCMP titration as compared to conventional titration methods [32].

Additionally, there is a controversy in the literature regarding the possible role of vertical opening. Each MAD has a given material thickness due to its construction features causing the mandible to be positioned simultaneously in a more caudal direction, resulting in an increase in the inter-incisal distance or so-called vertical dimension [35,36,37,38,39].

## 3. Side Effects

Mild and transient side effects are commonly reported in the initial period of MAD therapy. Short-term side effects include dry mouth, excessive salivation, tooth discomfort, muscle tenderness, temporomandibular joint pain, myofascial pain, and gum irritation [40,41]. These symptoms are mostly temporarily, generally resolving within days to weeks with regular use and appropriate adjustment of the device. However, sometimes these symptoms are more severe and continuous, resulting in cessation of the therapy. The major long-term adverse effects are occlusal changes with prolonged MAD use, but these changes have not been reported as being related to treatment withdrawal [42,43,44,45,46,47,48]. Conversely, skeletal or postural changes were negligible.

## 4. Effectiveness and Health Outcomes

MAD therapy for OSA does not eliminate the underlying causes of upper airway collapsibility but it is a lifelong treatment that prevents upper airway obstruction mechanically by protruding the mandible in order to decrease upper airway collapsibility and decrease the severity of OSA. Taking into account that even the most efficacious medical device is only effective when it is appropriately used, the overall therapeutic outcome can only be assessed when considering both the treatment’s efficacy as well as the patient’s adherence [49,50].

### 4.1. Efficacy

The efficacy of a given treatment modality gives us an idea of how good the therapy works when it is appropriately used. In sleep apnea, the efficacy of a therapy is expressed as a decrease in the apnea/hypopnea index (AHI). In general, MAD therapy reduces snoring and improves polysomnographic parameters like the AHI compared to placebo devices [26,51,52,53,54,55,56]. In general, approximately one-third of patients under MAD therapy show a complete resolution of the OSA disease obtaining an AHI < 5/h under MAD therapy, another third of patients showing a decrease in AHI with 50% or more while the last one-third of patients only showed a negligible improvement in OSA severity [57].

Overall, it has been reported that both CPAP and MAD therapy reduces the severity of OSA, however, CPAP reduced OSA severity to a greater extent than MAD therapy according to all studies comprising of patients with mild to severe OSA [58,59,60,61].

### 4.2. Adherence

The removable nature of an MAD warrants the assessment of its use and compliance in the treatment of OSA. Subjective self-reported adherence to MAD therapy is reported to be good, and in general greater compared with CPAP [14,62,63,64] although one study reported similar preference towards both treatment modalities [60] and one study documented a preference towards CPAP [61]. Furthermore, it is stated that adherence rates tend to decline over time [40,59]. The main reported reasons for discontinuation of MAD therapy include the presence of self-perceived side effects like excessive salivation, xerostomia, tooth and gingival discomfort and temporomandibular joint discomfort [40,41], and self-appreciated lack of efficacy. This emphasizes the patient’s perception of the treatment [40,65].

However, until recently, compliance data for MAD therapy have been limited to subjective self-reported use because of the lack of an objective compliance measurement in daily clinical practice. Caution should be taken when interpreting subjective compliance data since compliance studies in CPAP patients emphasize that they overestimate the objectively measured use by up to one hour [66].

Temperature-sensitive microsensors can be used to objectively measure MAD use. This technology was first used in the orthodontic treatment with removable oral appliances [67,68] and its application has now been introduced in sleep medicine.

The first report on the intraoral recording of MAD compliance during sleep was published by Lowe et al. [69]. The compliance monitor consisted of a ceramic thick-film hybrid with a memory system and temperature sensor which would monitor wear time based on the temperature measured above 31 °C. Objective MAD use was found to be 6.9 h/night over a two-week time span in eight patients. Nevertheless, several problems were reported with the compliance monitors that were used, including the damaging effect of saliva, heat intolerance of the electronic components, and energy consumption over a long period of trial time [69]. Inoko et al. reported on the assessment of the cytotoxicity of a temperature data logger in six OSA patients during 1 month [70]. In that study, the surface of the temperature data logger was coated with a temporary sealing material to prevent contact with oral mucosa [70]. A limitation of that specific study was the dimensions of the temperature data logger, which had a diameter of 17.4 mm, a thickness of 5.9 mm, and a weight of about 3.3 g. It is possible that the technical problems limiting the use of compliance monitors in both those previous studies [69,70] impeded the availability of objective measurement of MAD compliance in the field of sleep medicine until several years ago. Vanderveken et al. [50] recently showed that it is safe and feasible to measure the compliance objectively during MAD therapy using a microsensor thermometer embedded into the MAD. In that study, the objective MAD use was 6.7 ± 1.3 h/night with a regular user rate of 84% over a 3-month period [50]. At the 1-year follow-up, the discontinuation rate was 9.8%. The objective mean use rate was 6.4 ± 1.7 h/night at the 1-year follow-up in continuing users, with a regular use rate of 89% [71].

Using objective compliance measurements, a more pronounced decrease in complaints of socially disturbing snoring during MAD therapy was significantly correlated with better compliance during MAD therapy while the presence of dry mouth correlated with a lower compliance [72].

Currently, to our knowledge, there are three commercially available microsensors that could be applied in MAD therapy for the treatment of OSA: TheraMon (IFT Handels- und Entwicklungsgesellschaft GmbH, Handelsagentur Gschladt, Hargelsberg, Austria), Air Aid Sleep (AIR AID GmbH & Co KG, Frankfurt, Germany), and DentiTrac (Braebon Medical Corporation, Kanata, Canada). Kirshenblatt et al. [73] tested the accuracy of the three commercially available microsensors (TheraMon, AirAid and DentiTrac) in vitro using a water bath (34–37 °C) to simulate MAD wear time. The TheraMon microsensor was accurate during both short and long durations of simulated MAD wear. The AirAid sensor significantly underestimated MAD use during short durations of simulated MAD use with 3.67 ± 9.34 min/day, whereas the DentiTrac microsensor overestimated MAD use with 8.34 ± 3.62 min/day during short durations and with 3.53 ± 2.42 min/day during long durations of simulated MAD use. However, the under- or overestimations appear to be not clinically relevant.

### 4.3. Overall Clinical Effectiveness

**Figure 2:**
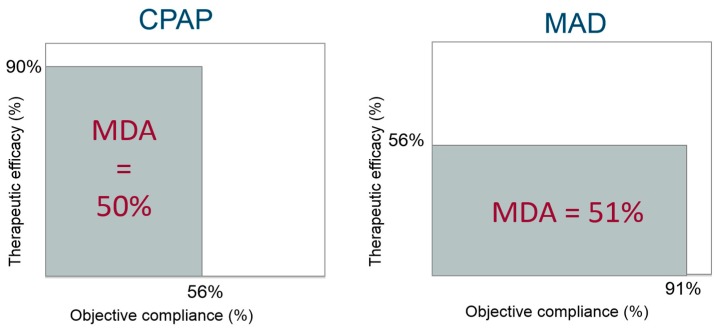
The mean disease alleviation (MDA) for continuous positive airway pressure (CPAP) vs. MAD therapy.

The mean disease alleviation (MDA) can be calculated as a combined function of efficacy and compliance and is a measure of the overall therapeutic effectiveness [49,50]. The MDA is equal to the surface area of the rectangle for which the length is given by the adjusted compliance, and the height is given by the therapeutic efficacy (AHI baseline minus AHI with therapy applied, expressed in percentage), Figure 2.

In the study of Vanderveken et al. [50], the mean AHI under MAD decreased with 56.0% compared to baseline with an adjusted compliance (objective MAD use adjusted for the total sleep time) of 91%. Consequently, the MDA was 51% (right panel of Figure 2). At the 1-year follow-up, similar results were obtained with an MDA of 55% [71].

Overall, the therapeutic effectiveness of MAD therapy is characterized by a suboptimal efficacy favored by a high compliance. Comparing these figures for CPAP versus MAD on therapeutic effectiveness supports the superiority of CPAP in efficacy in terms of reducing apnea severity but compromised by its lower compliance [74], resulting in a similar adjusted effectiveness as compared with MAD therapy (Figure 2) [58,75,76,77].

### 4.4. Health Outcomes

In a systematic review and recent meta-analysis, it has been shown that MAD therapy has a positive but minor effect on both systolic and diastolic blood pressure [78,79]. Studies comparing MAD with CPAP therapy show that both therapies are equally effective in reducing blood pressure and in reducing cardiovascular death [58,61,80,81,82]. This might be explained by the greater efficacy of CPAP being offset by inferior compliance relative to MAD, resulting in similar clinical effectiveness [58].

## 5. Patient Selection

Although MAD therapy significantly reduces OSA severity in the majority of patients, around one-third of patients only showed negligible improvement in OSA severity [57]. Therefore, patient selection and an individually tailored treatment plan is a key issue in MAD therapy and other non-CPAP treatment modalities. In the literature, it is described that MAD therapy is more likely to be successful in younger female patients [65], with lower body mass index [83], a smaller neck circumference [61] and less severe sleep apnea [26,65,84].

Ideally, however, patient selection for MAD therapy should be based on validated prospective elements.

Current predictive approaches for the different OSA therapies rely on the heterogeneity of the disorder and the variability in OSA pathophysiology between patients. Patients with mild upper airway collapsibility and low loop gain, referring to a more stable ventilatory control system, are more likely to benefit from MAD therapy [85,86].

Regarding anatomical phenotypes: cephalometric evaluations of morphological variables are inconsistent in predicting treatment success with MAD therapy [87].

Drug-induced sleep endoscopy (DISE) is a procedure that enables a dynamic evaluation of the localization and pattern of upper airway collapse [88]. This technique has shown its value in optimizing the selection of patients for surgical interventions of the upper airway [89]. In addition, it has been suggested as a valuable prognostic indicator of successful MAD therapy in the individual patient [90,91]. It is known that MAD treatment response is specifically related to the site, degree, and pattern of upper airway collapse as assessed during DISE. Based on the results of a recent prospective trial, tongue base collapse is a beneficial DISE phenotype and both complete concentric collapse at the level of the palate and complete laterolateral collapse of the oropharynx are adverse DISE phenotypes towards MAD treatment outcome [92].

Furthermore, during the DISE procedure, a so-called chin-lift maneuver can be performed, whereby the mandible is actively guided forward by grasping it and advancing it to a maximal protruded position. A possible criticism on the chin-lift maneuver is that it is a non-reproducible and, in most non-sedated patients, an unrealistic mandibular protrusion [93]. The use of a simulation bite in the maximal comfortable protrusion during the DISE procedure is a reproducible method to mimic mandibular protrusion. To make the simulation bite, the patient is asked to protrude the mandible maximally followed by a slow retraction of the mandible until a position is reached that the patient describes as the maximal comfortable protrusive position. This position is then transferred to a registration fork covered. The use of this simulation bite during DISE is found to be effective in predicting the therapeutic outcome of MAD therapy [93,94].

Recently, a “remotely controlled mandibular positioner” (RCMP) was applied in overnight polysomnography (PSG) to prospectively determine the optimal mandibular protrusion for MAD treatment in individual patients and to prospectively predict MAD treatment outcome [24,32]. By progressively protruding the mandible during a single-night sleep study, the mechanical action of the jaw advancement on the airway of the patient is simulated. It is shown that such a mandibular titration study with RCMP tends to predict therapeutic outcomes with MAD with significant accuracy [24]. However, this is a time-consuming and labor-intensive procedure, limiting routine clinical use. In order to avoid this, Kastoer et al. [95] recently showed that the use of an RCMP during a 30-min DISE is feasible and that it allows for the determination of the target mandibular protrusion. Nowadays, a feedback-controlled mandibular positioner identifies respiratory events in real-time was developed for use during a home-based sleep study (the “home-RCMP”) [96], protruding the mandible according to predetermined algorithms showing promising results.

## 6. Combination Therapy

In patients with limited mandibular protrusion, the addition of a tongue bulb may provide a better treatment effect, however, convenient appliance designs for this combination approach are needed [64].

In a retrospective analysis on patients undergoing MAD therapy, it was described that one-third of patients under MAD therapy have a residual positional OSA, defined as having twice as many respiratory events in the supine sleeping position compared to the non-supine sleeping position [97]. Patients with positional OSA can benefit from positional therapy, aimed at preventing sleep in the supine position. In Cartwright et al. [98], the efficacy of combining a posture alarm, which gives an auditory beep when in the supine position, and a tongue retaining device (TRD) was described. Patients were assigned to either therapy with the posture alarm, the TRD, or combination therapy with the posture alarm and the TRD. The results of that study suggested that the combination of TRD and positional therapy is better than one of the treatment modalities alone [98]. In a more recent study by Dieltjens et al. [99], the efficacy of combination therapy of a chest-worn vibrational alarm with MAD therapy was evaluated. The results indicate that combination therapy of MAD and positional therapy leads to a higher therapeutic efficacy in patients with a residual positional OSA under MAD compared to one of the treatment modalities alone [99]. These findings suggest that when patients are unsuccessfully treated with MAD treatment, the presence of the positional OSA should be checked and combination therapy could be suggested in eligible patients.

## 7. Conclusions

Oral appliance therapy is increasingly prescribed as a non-invasive treatment option for patients diagnosed with OSA. The recommended type of oral appliance is the custom-made, titratable mandibular advancement device allowing for gradual mandibular protrusion. Recent studies have shown comparable health outcomes with CPAP and MAD treatment, despite greater efficacy of CPAP in reducing OSA severity. This likely reflects greater nightly adherence to MAD compared to CPAP therapy. In unselected OSA patients, MAD therapy reduces OSA severity in the majority of these patients leaving about one-third with a negligible improvement. Therefore, the selection of appropriate candidates for MAD therapy is an ongoing item in order to increase the overall efficacy of the therapy. So far, several prediction tools have been proposed but currently, there is no validated method that can achieve a prospective up-front selection of the ideal candidate for MAD therapy in an accurate and reliable way.
